# CASynergy: A causal attention model for interpretable prediction of cancer drug synergy

**DOI:** 10.1371/journal.pcbi.1013567

**Published:** 2025-10-15

**Authors:** Haitao Li, Long Zheng, Lei Li, Yiwei Chen, Junjie Li, Chunhou Zheng, Yansen Su

**Affiliations:** 1 Key Laboratory of Intelligent Computing & Signal Processing, School of Artificial Intelligence, Anhui University, Hefei, Anhui, China; 2 Institute of Artificial Intelligence, Hefei Comprehensive National Science Center, Hefei, Anhui, China; 3 School of Internet, Anhui University, Hefei, China; Xinjiang Technical Institute of Physics and Chemistry, CHINA

## Abstract

Cancer drug combination therapies offer a promising strategy to overcome resistance and improve treatment efficacy, but identifying synergistic drug pairs is challenging due to complex biological interactions and tumor heterogeneity. Current machine learning algorithms for drug synergy prediction primarily rely on large-scale, multimodal datasets, yet suffer from critical limitations including poor interpretability, difficulty distinguishing causative biological relationships from correlations, and inadequate modeling of cancer-specific molecular interactions. To address these challenges, we propose CASynergy (Causal Attention and Cross-attention Synergy), a novel deep learning model for predicting cancer drug synergy that addresses limitations of prior approaches in accuracy and interpretability. CASynergy introduces a causal attention mechanism to distinguish true causal genomic features from spurious correlations, cell line-specific gene network construction to capture the unique molecular context of each cancer cell line, and a cross-attention module to integrate drug molecular features with cell line gene expression profiles. These improvements allow CASynergy to clearly identify significant drug-gene interactions and provides interpretable insights into why a combination is predicted to be synergistic. Experiments on two benchmark datasets (DrugCombDB and Oncology-Screen) suggests that CASynergy outperformed five state-of-the-art models. CASynergy offers a better and more reliable way to predict effective drug combinations. It works well across different cancer types and is easier to understand, which is important for personalized cancer treatment and finding new drugs.

## Author summary

Cancer often requires treatment with multiple drugs simultaneously to effectively combat resistance and improve patient outcomes. However, identifying which drug combinations work best together remains challenging because of the complex and diverse nature of tumors. To address this, we developed a new computational approach called CASynergy. Unlike traditional methods, CASynergy can clearly distinguish which genetic features are truly driving drug responses from those that are merely coincidental. This capability greatly enhances the accuracy and reliability of predicting effective drug combinations. Additionally, CASynergy uses detailed biological knowledge to build unique gene interaction maps for each cancer cell type, allowing our model to capture the individual characteristics of different tumors accurately. We tested CASynergy using well-established datasets and found it consistently outperformed existing methods. Moreover, it clearly shows researchers how different drugs affect specific genes, providing valuable insights into why certain drug combinations are effective. Our method thus not only improves prediction accuracy but also helps researchers and clinicians better understand drug interactions at the molecular level. Ultimately, this can guide more personalized and effective cancer treatments.

## 1 Introduction

Cancer, as one of the leading diseases threatening human health, continues to exhibit high incidence and mortality rates. Drug therapy is crucial in cancer treatment; however, monotherapy often faces challenges due to limited efficacy and drug resistance. Consequently, researchers are committed to exploring combination therapies to improve treatment outcomes, making the discovery of synergistic drug combinations a key focus in modern pharmacology and cancer therapy [1].

Traditional screening methods, such as high-throughput screening, have facilitated the discovery of novel drug combinations to some extent, but they are time- consuming, costly, and limited in identifying effective drug combinations [2]. In recent years, advances in machine learning have provided powerful alternatives to accelerate this process. Traditional machine learning methods, such as Support Vector Machines (SVM) [3]and Random Forests [4,5], rely on data including the physicochemical properties of drugs, drug-target interactions (DTI), and gene expression profiles of cell lines.While these methods perform well in predicting the effects of single drugs, their predictive capabilities are constrained by the complexity of drug combinations. Additionally, traditional methods often require manual feature engineering, making it difficult to effectively capture the hidden nonlinear relationships in high-dimensional data [6].

In contrast, deep learning models, with their ability to learn complex patterns from large datasets, have shown significant potential in predicting drug synergy, particularly in the field of anti-cancer therapies [7]. Deep learning approaches are primarily categorized into feature concatenation-based models and graph structure-based models. Feature concatenation- based models extract multimodal features, such as molecular characteristics of drugs and genomic data of cell lines, and concatenate these features into high-dimensional inputs for deep neural networks to predict synergy. For example, DeepSynergy [8] and MatchMaker [9] utilize fully connected neural networks to learn complex patterns from concatenated molecular and cell line features to predict drug synergistic effects. The advantages of these models lie in their simplicity and rapid adaptability to various data types. However, existing methods heavily rely on large-scale input data and complex feature extraction processes, which hinder their ability to effectively identify key causal features essential for drug synergy prediction. Furthermore, these methods often use simple concatenation or independently process drug features and gene expression data, limiting their capability to capture the subtle yet critical interactions between drugs and cell lines. Moreover, these models are often perceived as “black boxes,” lacking interpretability, which limits their clinical application and trustworthiness.

To better model the complex interactions among drugs, proteins, and cell lines, researchers have proposed graph structure-based models. These models use Graph Neural Networks (GNNs) [10] or knowledge graph embeddings [11] to integrate drug molecular structures, target information, and the biological characteristics of cell lines into graphs, thereby capturing higher-order interaction information. For instance, GraphSynergy [12] constructs a drug-protein- cell interaction network and employs Graph Convolutional Networks (GCNs) to capture network topology information, thereby enhancing the accuracy of drug synergy predictions. Similarly, KGANSynergy [13] predicts drug synergy by leveraging knowledge graphs and attention mechanisms to analyze relationships among drugs, targets, and cell lines, using entity embeddings and phenotypic features to represent drug-drug-cell triplets. HypergraphSynergy [14] casts drug–drug–cell line triplets as hyperedges and enhances generalization via an auxiliary similarity-network reconstruction task, achieving strong results and applicability to unseen combinations/cell lines. SDCNet [15] leverages a relational graph convolutional network with attention to learn both common and cell line-specific patterns within a unified model and reports robustness to new cell lines. While these methods demonstrate excellent predictive accuracy and biological interpretability, they fail to adequately consider disease-specific features, which may limit their predictive performance across diverse datasets and in personalized medicine.

In summary, current cancer drug combination synergy algorithms have the following limitations: first, existing deep learning models depend heavily on large amounts of input data and complex feature learning, making it difficult to effectively identify and utilize key causal features, and are viewed as “black boxes” lacking interpretability. Second, in cancer treatment, drug effects vary significantly across different cell lines or cancer types, and traditional models using a unified framework for prediction cannot effectively capture these molecular mechanism differences. Third, existing studies have not sufficiently integrated drug combination features with cell line features, failing to comprehensively reflect the impact and relationship of drug combinations on cell lines.

To address these limitations, we propose the Causal Attention and Cross-attention Synergy (CASynergy) model. CASynergy constructs cell line-specific networks and incorporates causal inference theory to achieve more stable, accurate, and interpretable predictions of synergistic drug combinations across different cancer types and datasets. The main innovations of the CASynergy model include:

1. A novel causal attention mechanism significantly enhances drug combination prediction by explicitly distinguishing causal features from non-causal noise, allowing the model to accurately capture crucial causal patterns. Unlike traditional correlation-based methods, this approach effectively identifies and mitigates confounding factors within causal pathways, thereby greatly improving the model’s generalization capability on out-of-distribution (OOD) datasets and ensuring robust predictive performance in diverse clinical scenarios [16].
2. By integrating cell line-specific feature topology networks and a sophisticated cross-attention mechanism, CASynergy precisely captures molecular heterogeneity and subtle interactions between drugs and cell lines. The cell line-specific networks leverage prior biological knowledge to construct unique gene association structures for each cell line, facilitating personalized modeling of drug synergy. Concurrently, the cross-attention mechanism seamlessly merges multiple data modalities, such as drug molecular features and gene expression profiles, significantly enhancing the model’s granularity and prediction accuracy.
3. Experimental results on the DrugCombDB and Oncology-Screen datasets demonstrate that the CASynergy model outperforms existing advanced algorithms in terms of accuracy, robustness, and interpretability. Particularly, CASynergy has excellent interpretability, allowing clear explanations of how specific drug combinations affect gene expression profiles. This helps researchers understand the biological mechanisms behind drug synergy predictions and provides insights for clinical decisions and personalized cancer treatments.

## 2 Results

**Fig 1:**
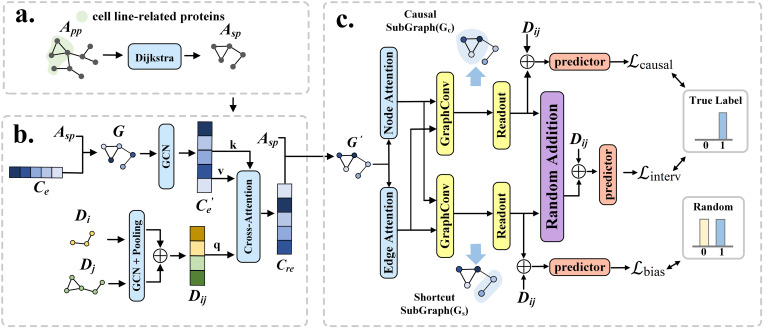
CASynergy algorithm workflow. **(a)** Cell line-specific gene network extraction module: Using the expression data of key genes in the cell line ${𝐜}^{\left(𝕚\right)}$ and the PPI network ${𝐀}_{pp}$, a disease-specific topological network is constructed; **(b)** Drug and cell line feature extraction and fusion module: The fusion of drug combination features and disease cell line features forms a semantic network; **(c)** Drug combination synergy prediction module based on causal attention learning: By combining the topological and semantic networks, and utilizing a causal inference-based attention mechanism, the synergy of drug combinations is predicted.

To improve the accuracy and interpretability of drug combination synergy prediction, we propose an algorithmic framework called Causal Attention and Cross-attention Synergy (CASynergy), as shown in Fig 1. The CASynergy algorithm integrates the molecular features of drug combinations with disease cell line-specific features to predict the synergy score of the drug combination for cancer treatment targeting the specific cell line. This approach enables the identification of drug combinations that are specifically effective for treating a particular disease. CASynergy consists of three modules: 1) the cell line-specific gene network extraction module, 2) the drug and cell line feature extraction and fusion module, and 3) the drug combination synergy prediction module based on causal attention learning.

### 2.1 Method comparison

To validate the effectiveness of CASynergy, we compared it with several mainstream drug synergy prediction methods: Random Forest, DeepSynergy [8], DeepDDS [17], GraphSynergy [12],and KGANSynergy [13].

**Random Forest**: A traditional machine learning method that utilizes drug molecular fingerprints and cell line gene expression information as inputs to predict drug synergy through an ensemble of decision trees (Random Forest).

**DeepSynergy** [8]: A deep learning-based drug synergy prediction model that employs neural network architectures such as convolutional layers to extract features from drug chemical structures and genetic information, modeling synergistic effects.

**DeepDDS** [18]: A deep model combining graph neural networks and attention mechanisms to predict drug synergy by aggregating molecular graphs and gene expression information, enabling high-level integration of molecular structures and cell line features.

**GraphSynergy** [12]: Utilizes spatial graph convolutional networks and attention mechanisms to encode high-order structural information of drug and cell line protein modules, thereby enriching entity embedding representations.

**KGANSynergy** [13]: Leverages knowledge graph structures and multi-head attention mechanisms to integrate multi-source neighbor node information during hierarchical propagation, enhancing the expressive power for drug synergy prediction.

**Fig 2:**
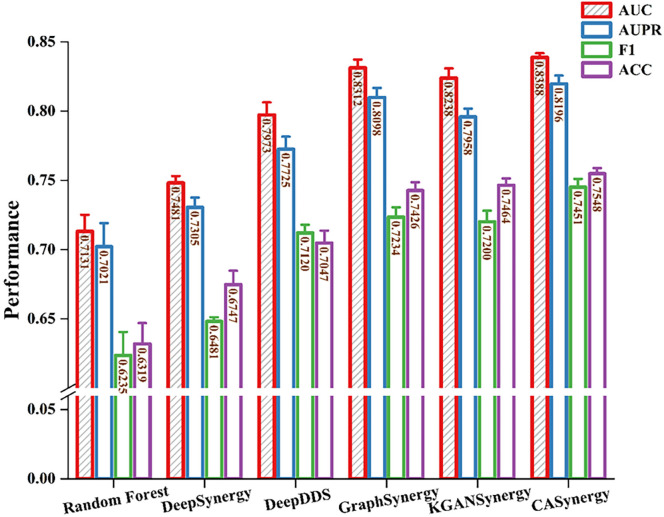
Comparative experimental results on DrugCombDB.

**Fig 3:**
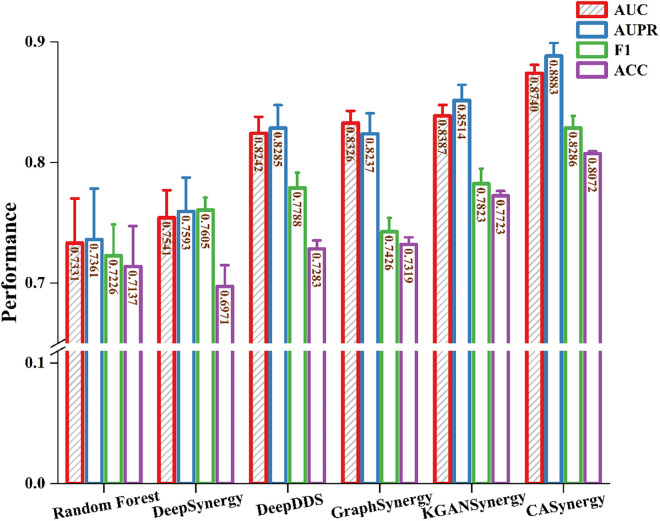
Comparative experimental results on oncology-screen.

These methods each have unique characteristics in drug synergy prediction, ranging from traditional machine learning to deep integrations of graph neural networks and knowledge graphs, providing diverse approaches for modeling synergistic interactions between drug combinations and cell lines. Tables 1 and 2 present the comparative experimental results on the DrugCombDB (Fig 2) and Oncology-Screen (Fig 3) datasets, respectively.

**Table 1:** Comparative experimental results on drugcombDB.

Dataset	AUC	AUPR	F1	ACC
Random Forest	0.7131 ± 0.012	0.7021 ± 0.017	0.6235 ± 0.017	0.6319 ± 0.015
DeepSynergy	0.7481 ± 0.005	0.7305 ± 0.007	0.6481 ± 0.003	0.6747 ± 0.010
DeepDDS	0.7973 ± 0.009	0.7725 ± 0.009	0.7120 ± 0.006	0.7047 ± 0.009
GraphSynergy	0.8312 ± 0.006	0.8098 ± 0.007	0.7234 ± 0.007	0.7426 ± 0.006
KGANSynergy	0.8238 ± 0.007	0.7958 ± 0.006	0.7200 ± 0.008	0.7464 ± 0.005
**CASynergy**	**0.8388 ± 0.003**	**0.8196 ± 0.006**	**0.7451 ± 0.006**	**0.7548 ± 0.004**

**Table 2:** Comparative experimental results on oncology-screen.

Dataset	AUC	AUPR	F1	ACC
Random Forest	0.7331 ± 0.0371	0.7361 ± 0.0421	0.7226 ± 0.0261	0.7137 ± 0.0335
DeepSynergy	0.7541 ± 0.0228	0.7593 ± 0.0281	0.7605 ± 0.0103	0.6971 ± 0.0176
DeepDDS	0.8242 ± 0.0137	0.8285 ± 0.0190	0.7788 ± 0.0127	0.7283 ± 0.0070
GraphSynergy	0.8312 ± 0.0102	0.8098 ± 0.0170	0.7426 ± 0.0113	0.7319 ± 0.006
KGANSynergy	0.8387 ± 0.009	0.8514 ± 0.013	0.7823 ± 0.0126	0.7723 ± 0.007
**CASynergy**	**0.8740 ± 0.004**	**0.8883 ± 0.006**	**0.8286 ± 0.0070**	**0.8072 ± 0.006**

From Table 1, it is evident that CASynergy achieved the highest AUC (0.8388) and AUPR (0.8196) on the DrugCombDB dataset, showing significant improvements over other methods. This indicates that CASynergy possesses higher predictive stability in distinguishing between positive and negative samples and balancing precision and recall. The F1 score (0.7451) and ACC (0.7548) also reached the highest levels. Overall, CASynergy maintains stable performance on large-scale and highly heterogeneous drug synergy data, demonstrating its strong generalization capability in integrating molecular graph structures with cell line-specific networks.

As shown in Table 2, on the smaller-scale Oncology-Screen dataset, CASynergy also outperformed other comparison methods with an AUC of 0.8740 and an AUPR of 0.8883, particularly showing an approximate 0.3% improvement in AUC, AUPR, and ACC compared to the latest KGANSynergy. Although both methods share similar concepts in leveraging knowledge graphs and multi-source information integration, CASynergy further enhances prediction accuracy and stability by employing a causal attention mechanism to more finely decouple and intervene on cell line-specific networks and molecular features. CASynergy also demonstrated notable advantages in the F1 score (0.8286) and ACC (0.8072), which measure the model’s comprehensive discrimination ability and overall prediction accuracy.

### 2.2 Performance evaluation by cold-start task

In practical cancer drug combination prediction, many new drug combinations are not sufficiently recorded in the existing datasets. Moreover, the heterogeneity of cancer causes significant differences in the responses of different cell lines to drugs, which poses challenges to model training and prediction. To validate the role of the CASynergy algorithm in actual drug combination prediction, we designed and conducted three cold-start experimental scenarios:

**Scenario 1**: Drug Cold-Start Task In this scenario, we randomly selected 80% of the drugs in the dataset as known drugs and used drug combinations composed of these known drugs as the training set. The remaining 20% of the drug combinations served as the test set to evaluate the model’s prediction ability when facing new drug combinations that have not appeared in the training set. This experiment was conducted five times.

**Scenario 2: Drug Combination Cold-Start Task.** This evaluation randomly samples 10% of drug combinations. For each sampled combination, we remove both constituent drugs from the entire training set (i.e., no training instance contains either drug), retrain the model on the remaining data, and evaluate only on that held-out combination. We repeat this procedure for all sampled combinations and report mean ± standard deviation across runs. This protocol rigorously assesses the model’s ability to generalize to genuinely unseen drug pairs.

**Scenario 3**: Cell Line Cold-Start Task In this scenario, the cell lines in the test set did not appear in the training set. The experimental setup was as follows: we selected 80% of the cell lines in the dataset as the training set, and the remaining 20% of the cell lines as the validation set. This experiment was also conducted five times.

We used the Oncology-Screen dataset for the experiments and compared CASynergy with several of the latest algorithms. The experimental results under the two cold-start scenarios are shown in Table 3.

**Table 3:** Performance comparison for drug and cell line and drug combination cold-start tasks.

Model	Scenario 1: Drug Cold-Start Task			Scenario 2: Drug Combination Cold-Start Task			Scenario 3: Cell Line Cold-Start Task		
	AUC	AUPR	ACC	AUC	AUPR	ACC	AUC	AUPR	ACC
DeepSynergy	0.673 ± 0.0121	0.5926 ± 0.0215	0.5847 ± 0.0114	0.6643 ± 0.0798	0.6668 ± 0.0705	0.5983 ± 0.0578	0.6528 ± 0.0324	0.6561 ± 0.0338	0.6196 ± 0.0247
DeepDDS	0.6566 ± 0.008	0.5872 ± 0.0178	0.582 ± 0.0133	0.5773 ± 0.0845	0.6158 ± 0.0726	0.5474 ± 0.0876	0.7204 ± 0.0062	0.7292 ± 0.0072	0.6720 ± 0.0076
GraphSynergy	0.6607 ± 0.044	0.7005 ± 0.0723	0.6028 ± 0.0279	–	–	–	0.7472 ± 0.0359	**0.7861 ± 0.0639**	0.6749 ± 0.0232
KGANSynergy	0.6607 ± 0.035	0.677 ± 0.0723	0.6403 ± 0.0327	–	–	–	0.5655 ± 0.0207	0.5520 ± 0.0232	0.6077 ± 0.0071
CASynergy	**0.7512 ± 0.0048**	**0.7154 ± 0.0153**	**0.7066 ± 0.004**	**0.6732 ± 0.0645**	**0.6745 ± 0.0783**	**0.7034 ± 0.0592**	**0.7500 ± 0.014**	0.7530 ± 0.0169	**0.7559 ± 0.0062**

In the drug cold-start task experiment, CASynergy significantly outperforms other algorithms in both AUC (0.7512) and AUPR (0.7154), especially compared to DeepSynergy (AUC: 0.673, AUPR: 0.5926). The AUC of CASynergy is about 11. 6% higher and the AUPR is about 20.7% higher. CASynergy shows greater stability and reliability. In the experiment of the cell line cold start task, CASynergy also performs excellently, with an AUC value of 0.75, which is approximately 14.8% higher than DeepSynergy’s 0.6528. The AUPR value of CASynergy is 0.753, about 14.8% higher than DeepSynergy’s 0.6561. In terms of ACC, CASynergy’s performance leads with an ACC of 0.7559, which is about 22% higher than DeepSynergy, demonstrating its ability to provide more accurate drug response predictions when handling cell line cold starts. In the drug combination cold-start task experiment, CASynergy outperforms the baselines across all metrics. It attains AUC 0.6732 and AUPR 0.6745. More importantly for classification, CASynergy achieves F1 0.7512 and ACC 0.7034, substantially exceeding DeepSynergy (0.6345/0.5983) and DeepDDS (0.6112/0.5474). These results show that CASynergy maintains competitive ranking performance while delivering significantly decision quality under the most demanding cold-start condition, underscoring its robustness and practical utility for predicting synergy of entirely unseen drug combinations.

In summary, the effectiveness of CASynergy in drug synergy prediction tasks has been thoroughly validated. It outperforms other latest comparative algorithms, particularly in cold-start tasks, showing significant improvements in AUC and AUPR. CASynergy demonstrates excellent adaptability under different data scales and feature distribution conditions, and its strong generalization capability allows it to effectively handle unseen drug combinations and cell lines. These results indicate that CASynergy provides more accurate and reliable predictions for drug combinations in real-world applications, showcasing its greater potential for both application and clinical value.

### 2.3 Ablation study

To investigate the influence of different modules on the performance of the CASynergy algorithm, we conducted an ablation study focusing on key components. Specifically, we constructed the following four model variants for comparison against the full CASynergy model:

- **w/o spp**: Does not use the cell-line-specific PPI network (spp). Instead, it directly adopts the original PPI edges as the cell line graph structure.
- **w/o CA fusion**: Does not employ the Cross-Attention fusion (CA fusion) mechanism to fuse the drug combination and cell line features; rather, it simply concatenates the two feature sets and feeds the result into downstream modules.
- **CASynergy-BLA**: cross-modal interactions are modeled via a bilinear scoring function
- **w/o cal**: Does not use causal attention learning (cal) to decouple the cell line graph structure; instead, it directly utilizes GCNs to obtain cell line embedding vectors for prediction.

**Fig 4:**
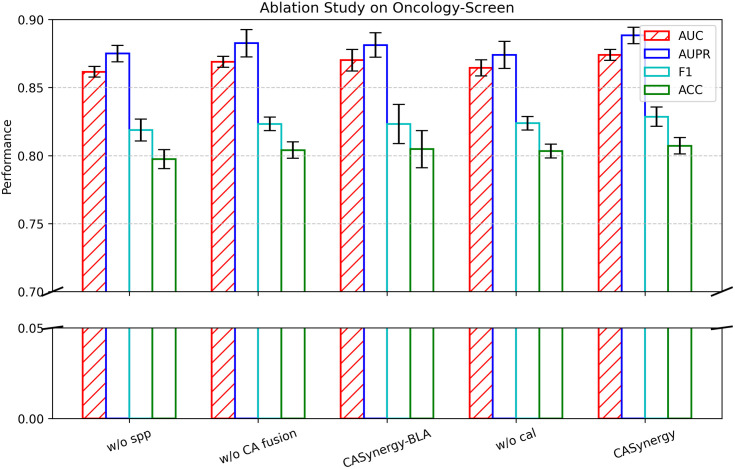
Ablation study on oncology-screen.

As shown in Table 4 and Fig 4, the cell-line-specific PPI network and the causal decoupling layer (cal) play crucial roles in the model. Removing the cell-line-specific PPI network leads to decreases in AUC, AUPR, and F1 (e.g., AUC drops from 0.8740 to 0.8616), suggesting that the cell-line-specific PPI network captures more pertinent protein and gene information for each cell line, thereby enhancing predictive accuracy. In contrast, employing only the raw PPI edges fails to adequately capture the unique gene interaction patterns of each cell line, resulting in diminished performance.

**Table 4:** Ablation study on oncology-screen.

Dataset	AUC	AUPR	F1	ACC
**w/o spp**	0.8616 ± 0.004	0.875 ± 0.006	0.8188 ± 0.008	0.7973 ± 0.007
**w/o CA fusion**	0.8689 ± 0.004	0.8826 ± 0.010	0.8233 ± 0.005	0.8040 ± 0.006
**CASynergy-BLA**	0.8701 ± 0.0079	0.8813 ± 0.0089	0.8232 ± 0.0143	0.8047 ± 0.0136
**w/o cal**	0.8645 ± 0.006	0.8740 ± 0.010	0.8238 ± 0.005	0.8033 ± 0.005
**CASynergy**	**0.8740 ± 0.004**	**0.8883 ± 0.006**	**0.8286 ± 0.0070**	**0.8072 ± 0.006**

Eliminating the cross-attention module causes the AUC to drop from 0.8740 to 0.8689 and AUPR to drop from 0.8883 to 0.8826, demonstrating that cross-attention allows for deeper integration of drug and cell line features when modeling differences in cell line targets under distinct drugs. Although this decrease is less pronounced than with other modules, the results still reveal the critical role cross-attention plays in further boosting the model’s performance.

Finally, omitting causal attention learning lowers the AUC from 0.8740 to 0.8645, indicating that causal attention learning helps identify genes that exhibit a true causal relationship with the labels while suppressing purely correlational genes or noise, thereby enhancing model robustness and interpretability.

Overall, the ablation study highlights the pivotal contributions of the cell-line-specific network, cross-attention mechanism, and causal attention learning to CASynergy. Only by jointly employing these components can one maximize drug synergy prediction performance and achieve high model interpretability.

### 2.4 Interpretability analysis

In practical applications, understanding how synergistic drug combinations effectively treat specific diseases generally involves revealing a crucial question: through which critical genes or biological processes do drug combinations exert their therapeutic effects? Addressing this question is central to predicting drug synergy and is a critical step in studying drug therapeutic mechanisms. To better address this question and provide clear interpretability analysis, the CASynergy algorithm integrates causal inference mechanisms and attention mechanisms, effectively identifying the specific impact of drug combinations on gene expression in cell lines, thus providing intuitive and reliable biological explanations.

CASynergy utilizes causal inference methods to analyze the effect of drug combinations on gene expression in cell lines. One of the core interpretability tools provided by CASynergy is the causal subgraph soft mask matrix $M_{x}$. By analyzing this matrix, we can clearly identify key genes whose expression levels are significantly altered by drug combinations in cell lines and subsequently discern potential biological mechanisms based on the analysis of these critical genes.

To validate the interpretability of the CASynergy algorithm, this study identifies key therapeutic genes by analyzing the attention score soft mask matrix $M_{x}$ generated by CASynergy. Firstly, the matrix $M_{x}$ of a specific synergistic drug combination was extracted from a trained CASynergy model. Genes ranking in the top 25% based on attention scores were defined as key genes. Then, the cumulative number of key genes across all synergistic drug combinations for the given cell line was calculated. Subsequently, the top 20% of genes from this cumulative distribution were selected as critical genes associated with synergistic drug combinations for that cell line. A KEGG biological pathway enrichment analysis was then performed to identify biological pathways associated with these key genes.

**Fig 5:**
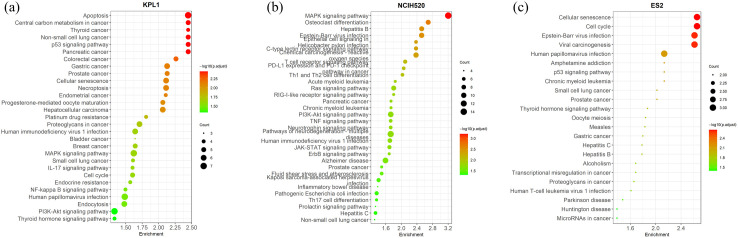
Biological pathway enrichment analysis for key genes identified by the CASynergy algorithm in different cancer cell lines. **(a)** Breast cancer cell line KPL-1. **(b)** Lung squamous cell carcinoma cell line NCI-H520. **(c)** Ovarian clear cell carcinoma cell line ES2.

In this study, we exemplify this approach using the KPL-1 (human breast cancer cell line), NCI-H520 (human lung squamous cell line), and ES2 (human ovarian clear cell carcinoma cells). Our experiments demonstrate that the cumulative number of key genes in the cell lines follows a power-law distribution (as shown in Fig S1), indicating that changes in gene expression induced by drug combinations predominantly concentrate on a few critical genes that decisively influence therapeutic outcomes. Subsequently, we performed interpretability analysis using Enrichr (https://maayanlab.cloud/Enrichr/) [19], selecting pathways with adjusted p-values less than 0.1 as critical pathways for enrichment analysis; results are depicted in Fig 5.

For the breast cancer cell line KPL-1 (as shown in Fig 5(a)), apoptosis is a crucial process in cancer therapy. Drug combinations inducing apoptosis can effectively eliminate cancer cells by activating intrinsic and extrinsic apoptotic pathways. In breast cancer contexts, drugs that promote apoptosis may inhibit tumor growth by overcoming resistance to cell death. Research has shown that combining ruxolitinib and MK-2206 can regulate apoptotic pathways by dual inhibition of JAK2/STAT5 and PI3K/AKT signaling pathways, thus treating breast cancer [20]. Central carbon metabolism is essential for cancer cell metabolic reprogramming, involving key metabolic pathways such as glycolysis, TCA cycle, and pentose phosphate pathway. These pathways provide energy and biosynthetic precursors critical for rapid cancer cell proliferation and survival. Metabolic reprogramming is closely related to resistance to chemotherapy and radiotherapy.

Targeting these metabolic pathways can improve therapeutic effectiveness and overcome drug resistance [21]. In vitro studies demonstrated promising effects of metabolic inhibitors combined with chemotherapeutics. For example, combinations of CB-839 (a glutaminase inhibitor) and Oxamate (a lactate dehydrogenase inhibitor), as well as the triple combination of CB-839/Oxamate/D609 (phosphatidylcholine- specific phospholipase C inhibitor), induced significant cell death in breast cancer cell lines. These inhibitors also enhanced the efficacy of doxorubicin, potentially reducing patient exposure and cardiotoxicity while maintaining therapeutic effects [18]. The p53 tumor suppressor gene is pivotal in regulating the cell cycle and apoptosis in response to DNA damage. Loss of p53 function, common in cancers, contributes to tumorigenesis. Certain drug combinations can activate the p53 pathway, enhancing apoptotic responses and effectively inhibiting breast cancer cell growth [22]. For instance, combining Renieramycin M with doxorubicin upregulates p53 protein expression, promotes activation of downstream target genes, enhances cellular responses to DNA damage, and ultimately induces apoptosis. This synergy increases treatment sensitivity in breast cancer cells, offering a potential therapeutic strategy [23].

For lung squamous carcinoma (LUSC) cell line NCI- H520 (Fig 5(b)), numerous studies demonstrate enhanced therapeutic efficacy by drug combinations targeting the MAPK signaling pathway. Such combinations typically simultaneously target different nodes of the MAPK pathway, including upstream receptor tyrosine kinases (such as EGFR) and downstream kinases (such as MEK), achieving comprehensive tumor growth inhibition. For instance, in non-small cell lung cancer (NSCLC) models harboring MET exon 14 skipping mutations, coexisting KRAS activation or NF1 loss can hyperactivate RAS-MAPK signaling, limiting MET inhibitor efficacy. However, combining MET inhibitors (e.g., crizotinib) with MEK inhibitors (trametinib) can concurrently block reactivation of this pathway, thus overcoming resistance. LUSC patients often experience bone metastases, and osteoclast- mediated bone destruction can accelerate tumor progression. Studies indicate that targeting osteoclast-related pathways improves LUSC treatment outcomes. Denosumab, which blocks the RANK/RANKL pathway, inhibits osteoclast activity and bone destruction. Clinically, adding Denosumab to treatment significantly improves overall survival compared to traditional bisphosphonates [24]. Additionally, RANKL inhibition exhibits immune-regulatory effects. Preclinical studies by Ahern et al. demonstrated that combining anti-RANKL agents with anti- PD-1 immune checkpoint inhibitors increases tumor-infiltrating T cells and antitumor cytokines such as IFN-γ, significantly reducing tumor burden. In advanced NSCLC patients with bone metastases, combining Denosumab with PD-1/PD-L1 inhibitors improved response rates and progression-free survival without significant additional toxicity [25].

In ovarian cancer cell line ES2 (Fig 5(c)), cellular senescence and cell cycle pathways are critical targets of drug combinations. Classical chemotherapy drugs such as platinum agents and paclitaxel induce therapy-induced senescence (TIS). Studies in high-grade serous ovarian cancer mouse models with homologous recombination deficiency (HR-deficient, e.g., BRCA mutation) show that platinum chemotherapy efficiently induces cellular senescence [26]. These senescent cells activate the cGAS/STING pathway, inducing a limited senescence-associated secretory phenotype (SASP), recruiting immune cells, and enhancing sensitivity to immune checkpoint inhibitors. Tumors prone to senescence show better responses when chemotherapy is combined with immunotherapy (PD-1/PD-L1 antibodies). Additionally, topoisomerase inhibitors (such as doxorubicin, etoposide) induce senescence; subsequently adding the BCL-2/BCL- XL inhibitor ABT-263 (Navitoclax) selectively eliminates these cells, substantially reducing tumor volume [27]. This mechanism presents a potentially effective drug combination strategy for treating ovarian cancer.

Through the above experimental analyses, we demonstrated the interpretability of the CASynergy algorithm in predicting drug combination synergistic effects. Firstly, causal inference and attention mechanisms effectively identified the critical biological processes impacted by drug combinations in cell lines, elucidating molecular mechanisms underlying synergistic actions. Secondly, by utilizing the soft mask matrix $M_{x}$, we provided a deep understanding of how drug combinations achieve synergistic therapeutic effects through specific biological processes. This enhances model transparency, provides a theoretical foundation for subsequent experimental validation, and facilitates practical applications of the model.

### 2.5 Case study

To verify the adaptability and practical potential of the CASynergy model, we conducted predictions for novel drug combinations on the Oncology-Screen dataset. Specifically, we first constructed candidate drug combinations using the drug and cell line data from Oncology-Screen; then, the CASynergy model evaluated the synergy scores of these combinations in designated cell lines. We selected the top 100 drug combinations with the highest predicted synergy scores for in-depth analysis and found 14 synergy combinations supported by published literature (see Table 5).

**Table 5:** Fourteen highly predictive synergistic drug combinations in oncology-screen supported by literature.

Cell Line	Tissue	Drug 1	Drug 2	PMID
NCIH1650	Lung	PACLITAXEL	SUNITINIB	19031964
SKMES1	Lung	VINBLASTINE	SUNITINIB	26717366
NCIH460	Lung	ZOLINZA	GEMCITABINE	16153448
NCIH2122	Lung	ZOLINZA	DASATINIB	34224975
NCIH2122	Lung	ZOLINZA	SORAFENIB	22415798
KPL1	Breast	PACLITAXEL	LAPATINIB	34126209
KPL1	Breast	DOXORUBICIN	ZOLINZA	37716547
MDAMB436	Breast	LAPATINIB	BORTEZOMIB	20701607
ZR751	Breast	VINORELBINE	DASATINIB	29416938
ZR751	Breast	PACLITAXEL	ZOLINZA	22200869
SKMEL30	Skin	GELDANAMYCIN	SORAFENIB	20525756
LOVO	Large Intestine	ZOLINZA	BORTEZOMIB	19174560
LOVO	Large Intestine	METFORMIN	SORAFENIB	36304185
CAOV3	Ovary	METFORMIN	SORAFENIB	31256441

For instance, gemcitabine suppresses NF-κB activation induced by vorinostat and concurrently inhibits chromatin remodeling mediated by histone deacetylases, prompting apoptosis in NSCLC [28]. Paclitaxel, administered alone or in combination with other anticancer agents—particularly in HER2-negative breast cancer cells—exerts enhanced cytotoxicity through lapatinib-induced inhibition of ABC transporters, effectively treating both early and metastatic breast cancer [29,30]. Sorafenib, a multi-target kinase inhibitor, mainly acts on the RAF kinase pathway and vascular endothelial growth factor receptors; tanespimycin, as an Hsp90 inhibitor, affects key signaling proteins such as RAF-1. Their synergy arises from acting on distinct but interrelated signaling pathways, inhibiting tumor cell proliferation and angiogenesis, thereby providing a potentially effective therapeutic strategy [31]. Vorinostat(Zolinza) exerts its biological effects by inhibiting histone deacetylases, whereas bortezomib inhibits proteasome formation, affecting the cell cycle and apoptosis. Their combined application in colon cancer cells demonstrates a synergistic effect, significantly enhancing the inhibition of cell proliferation, underscoring the superior efficacy of their joint use [32].

These cases not only underscore the high predictive accuracy of CASynergy in determining synergistic drug effects but also highlight its capability in identifying biologically plausible drug combinations.

## 3 Discussion

In this study, we developed CASynergy, a computational model that significantly improves the prediction of synergistic cancer drug combinations. The model’s key contributions include the integration of causal inference and attention mechanisms to focus on truly influential molecular features, and the use of cell line-specific gene network topology to account for biological differences between cancer types. By combining causal attention (to filter out confounding signals) and cross- attention (to capture detailed interactions between drug properties and gene expression), CASynergy addresses major challenges in the field. These innovations enable CASynergy to deliver more stable and accurate synergy predictions across different datasets and conditions. Our experimental results demonstrate that CASynergy achieves superior performance on large-scale heterogeneous data, maintaining high predictive accuracy and generalization where previous models struggled. Notably, CASynergy attained the highest AUC and AUPR scores on both the DrugCombDB and Oncology-Screen benchmarks, reflecting its strong overall discrimination ability and balanced precision–recall performance. These findings confirm that the proposed model not only advances prediction accuracy but also offers greater interpretability and robustness, thereby contributing a valuable tool for personalized cancer therapy research. Compared to existing drug synergy prediction approaches, CASynergy offers marked improvements in accuracy, robustness, and interpretability.

While effective to an extent, such feature-concatenation models (including DeepSynergy and DeepDDS) often treat drug–gene interactions as a “black box,” making it difficult to discern which biological factors drive synergy. In contrast, CASynergy’s causal attention mechanism allows it to identify and weight only the truly causal genomic features for synergy, improving interpretability and out-of-distribution generalization.

Graph-based methods (including GraphSynergy and KGANSynergy) represent another advance, using biological interaction networks to inform predictions. These approaches improve prediction accuracy and provide some biological insights. Nonetheless, they do not fully incorporate disease- specific molecular contexts; for example, they treat all cell lines in a unified network without tailoring to individual genetic backgrounds. CASynergy distinguishes itself by constructing cell line-specific gene networks, effectively personalizing the interaction topology to each cancer type or cell line. This design captures critical differences in molecular pathways between cell lines, which prior models might overlook. As a result, CASynergy demonstrated stronger performance and stability across diverse contexts. It outperformed GraphSynergy and KGANSynergy on both benchmarks, with particularly notable gains on the Oncology-Screen dataset (improving AUC, AUPR, and accuracy by 0.3% over the best prior model, KGANSynergy).

Furthermore, in challenging cold-start scenarios where the model must predict synergy for new drugs or new cell lines not seen in training, CASynergy maintained high accuracy. CASynergy integrates advantages of previous deep learning and graph-based methods. It overcomes their limitations, providing a model with improved accuracy, better reliability on new data, and clearer interpretability.

In real-world settings, missing values in cell-line transcriptomic profiles commonly arise from assay dropouts and differences between platforms. Specifically, we randomly masked 10%, 20%, or 30% of genes in each cell line and used imputers the MICE package in R to perform mean imputation on the gene expression matrices. The imputed matrices were then evaluated with five-times five-fold cross-validation using the same training protocol as the main experiments (as shown in Table S4). CASynergy shows minimal performance degradation as the masking ratio increases from 0% to 30%. To further and more systematically examine how performance scales with data volume, we added a controlled data-efficiency study on Oncology-Screen. We created stratified training subsets containing 30%, 50%, and 70% of the data and kept the evaluation protocol identical to the main experiments (Table S5). This high robustness is likely due to the model’s causal inference framework. The model’s causal attention focuses on stable drug-gene interactions that exist across all cell lines, ignoring random associations that can be easily lost. Guided by the structure of cell-line-specific networks, its cross-modal attention mechanism aligns and combines strong signals within the same biological pathway, which helps to remove noise and fill in missing information. Furthermore, the causal design makes the model focus on key causal factors and uses redundant information from multiple sources (drug structure and gene expression), making it less sensitive to errors.

Despite its advancements,CASynergy has certain limitations that should be acknowledged. First, the model’s effectiveness relies on the availability of high-quality structured omics data and prior biological knowledge for each cell line. CASynergy uses predefined cell line-specific gene networks (e.g., protein–protein interaction or gene co-expression networks) to inform its feature topology. This reliance means that for cancer types or cell lines where such network data are incomplete or unavailable, the model’s performance may be impacted. Moreover, CASynergy currently focuses on transcriptomic gene expression and known gene interactions; other molecular factors (such as genetic mutations, epigenetic modifications, or proteomic changes) are not explicitly integrated, which may limit the model’s ability to capture all determinants of drug response. These limitations suggest that, although CASynergy represents a meaningful step forward, there is room for further refinement to enhance its general applicability and ease of use.

In the future, the CASynergy algorithm can be improved and expanded through the following aspects. An important future direction is the integration of multi-omics data to provide a more comprehensive view of tumor biology. Incorporating genomic mutations, copy number variations, proteomic and metabolomic profiles, or epigenetic markers alongside gene expression could allow the model to learn a richer representation of each cell line’s state. To assess the practical feasibility of this direction in our current setting, we performed a controlled multi-omics augmentation on the Oncology-Screen cohort. We matched CCLE mutation and copy-number (CNV) profiles to the same cell lines and compared four feature configurations for CASynergy: (i) gene expression only; (ii) expression + mutation; (iii) expression + CNV; and (iv) expression + mutation + CNV. All variants were capacity-matched and trained under identical protocols with five-times five-fold cross-validation. Expression-only already matched or slightly outperformed the multi-omics variants on AUC/AUPR/F1 (AUC 0.8740 ± 0.004, AUPR 0.8883 ± 0.006), while the triple-omics setting produced only a small ACC uptick (0.8098 ± 0.006) without consistent gains elsewhere (see S6 Table). These findings suggest that, at the current dataset scale and without pathway-level integration, most synergy-relevant variation is already encoded in transcriptomics, whereas naïvely concatenating mutation/CNV features may add redundancy and noise. Going forward, we will investigate principled integration strategies—e.g., pathway/module aggregation, shared–private factorization, and causal multi-view training on harmonized profiles—to more effectively exploit complementary omics in larger cohorts. Another important future direction is to expand the scope of CASynergy to handle a broader range of drug types and combination strategies. This could involve predicting synergies that include not only small-molecule drugs but also biologics (such as therapeutic antibodies), immunotherapies, or even combinations of more than two agents. Adapting the model to these more complex or diverse therapy regimens would increase its relevance to real-world cancer treatment scenarios. Moreover, developing simplified versions of CASynergy or user- friendly tools that highlight the key pathways and gene targets driving a predicted synergy can help oncologists and researchers trust and effectively use the model’s recommendations. In summary, the CASynergy approach can be further evolved into a powerful decision-support system for combination cancer therapy discovery and optimization.

## 4 Materials and methods

### 4.1 Datasets

This study systematically prepared datasets by constructing drug synergy datasets and cell line-related datasets to comprehensively evaluate the performance of CASynergy in predicting drug synergy under various cell line conditions.

**Drug synergy datasets** To evaluate the performance of CASynergy in the task of drug synergy prediction, we selected two publicly available datasets of varying scales and sources: DrugCombDB and Oncology- Screen (see Table 6). These datasets include experimental results such as drug combinations and their synergy scores, effectively demonstrating CASynergy’s adaptability under different scales and conditions.

**Table 6:** Details of DrugCombDB and oncology-screen datasets.

Dataset	Single Drugs	Cell lines	Drug Combination	
			Synergy	Non-synergy
Oncology-Screen	21	29	2257	1919
DrugCombDB	764	76	31684	37752

**DrugCombDB** [33]: DrugCombDB is a comprehensive drug combination dataset sourced from high-throughput screening experiments, multiple external databases, and relevant literature on PubMed. The DrugCombDB dataset used in this study comprises 69,436 drug combinations and their synergy scores, covering 764 drugs and 76 cancer cell lines.

**Oncology-Screen** [34]: The Oncology-Screen dataset originates from a large-scale tumor screening conducted by O’Neil et al., encompassing 4,176 drug combinations and their synergy scores, involving 21 drugs and 29 cancer cell lines.

In the experiments, drug combinations with synergy scores greater than 0 are considered positive samples with synergistic effects, while those with scores less than 0 are regarded as negative samples without synergistic effects. This classification facilitates the construction of a binary classification task for synergy prediction.

**Cell line-related datasets** To further explore the cell line-specific network information and gene expression differences in cancer cell lines, this study incorporates additional cell line-related data based on the drug synergy data. This integration aids CASynergy in better understanding and modeling the molecular-level differences among cell lines.

**Protein-Protein Interaction (PPI) Networks**: We utilized the ‘Human Protein-Protein Interaction (PPI) Network’ integrated by Cheng et al. [35], which consolidates interactions from 15 experimentally validated common databases. This network comprises 217,160 interactions among 15,790 protein nodes, providing the topological foundation at the gene-protein interaction level for this study.

**Cell Line-Protein Relationships**: Leveraging the cell line-protein relationship data collected by Yang et al. [12] from the CCLE [36] database, we mapped 18,022 genes to their corresponding proteins and associated them with 1,035 cancer cell lines. This mapping process facilitates the precise identification of active or key gene-protein interactions within each cell line, thereby establishing a data foundation for constructing cell line-specific gene networks.

**Cell Line Gene Expression Data**: Based on the cell line-protein relationships, we selected protein-coding genes relevant to the target cell lines and removed isolated gene nodes in the constructed specific PPI networks. The DrugCombDB dataset utilizes 7,217 genes, while the Oncology-Screen dataset employs 2,865 genes, with a certain degree of overlap between these gene sets. The gene expression data primarily originate from the CCLE and GEO databases (see S1 Table), and were standardized prior to use.

By integrating the aforementioned drug synergy data with cell line-related data, this study accurately characterizes the potential synergistic responses of different cell lines to drug combinations from multiple perspectives, including gene expression and cell line-specific network topology. This comprehensive data support underpins the construction of cell line-specific models within CASynergy.

### 4.2 Cell line-specific gene network extraction module

To better reveal the underlying molecular mechanisms of disease onset and progression, this study constructs cell line specific feature topological networks based on protein-protein interaction (PPI) relationships specific to each cell line (Fig 1(a)). Research has shown that although cancer cell lines of the same type may exhibit similarities in certain aspects, their protein interaction networks differ significantly. Therefore, mining specific gene network modules can provide a more effective basis for drug combination prediction.

Given a protein-protein interaction network (PPI) App=(V,E), where V represents the network nodes (proteins), and E represents the edges in the network, with an edge value of 1 if there is an interaction between two proteins, and 0 otherwise. For cell line 𝕚, the key target proteins $T^{\left(𝕚\right)}\subseteq V$ of the cell line are first identified based on the protein-cell line interaction relationship. These key proteins are used as seed nodes in the PPI network. Next, a connected subgraph with minimal total edge count is constructed to generate the cell line-specific PPI network structure by introducing additional protein nodes (i.e., intermediate nodes). Specifically, a heuristic method based on the shortest path is used to approximate the construction of the connected specific PPI network structure $A_{sp}^{\left(i\right)}$. The Dijkstra algorithm is employed to calculate the shortest paths between seed nodes. Then, intermediate nodes (non-seed nodes) from these shortest paths are gradually introduced to connect unconnected sub-networks. In this process, a greedy strategy is adopted, prioritizing the connection with the shortest total path length to minimize the total number of edges in the network. Through iterative optimization, the network connectivity is progressively improved, ultimately generating a connected, specific PPI network $A_{sp}^{\left(i\right)}$ for the cell line $c^{\left(𝕚\right)}$. This method is highly efficient when handling large-scale PPI networks and effectively preserves the key topological features of the network.

### 4.3 Drug and cell line feature extraction and fusion module

In order to investigate how drugs affect the expression of key protein-coding genes in cell lines, this study analyzes relevant features within the drug and cell line feature extraction and fusion module (Fig 1(b)). By examining the impact of drugs on gene expression in cell lines, it is possible to more accurately identify key genes jointly influenced by drug combinations at the molecular level, thereby improving the accuracy of synergy prediction and laying a foundation for interpretable analyses of drug mechanisms.

Specifically, based on the previously described cell-line-specific gene network extraction module, we first consider the key topological features of cell line i, denoted by $A_{sp}^{\left(i\right)}$, and their corresponding gene expression features $C_{e}^{\left(i\right)}$. We then construct a cell-line-specific key gene expression network $G^{\left(i\right)}=(C_{e}^{\left(i\right)},A_{sp}^{\left(i\right)})$, where $C_{e}^{\left(i\right)}\in R^{G_{n}\times \text{1}}$ represents the expression data of the key genes in the network, $G_{n}$ denotes the number of genes in the cell line, and $A_{sp}^{\left(i\right)}$ refers to the key topological features of cell linei.

Next, for the extraction of drug features, we use the molecular structural information of drugs. The molecular structure of a drug is transformed from its SMILES string into a molecular graph G=(X,A). Here, $X\in R^{n\times F_{d}}$ denotes the feature matrix of all atoms within the molecular graph, where n is the number of atoms, and $F_{d}$ represents the feature dimension of each atom. The adjacency matrix $A\in R^{n\times n}$ encodes the chemical bond relationships between atoms. We employ a Graph Convolutional Network (GCN) to process this molecular graph. The operation of the l-th GCN layer can be defined as:


$$H^{\left(l\right)}=relu\left(\hat{A}H^{\left(l-\text{1}\right)}W^{\left(l\right)}\right),$$ (1)

where $H^{\left(l\right)}$ represents the hidden feature matrix at layer l, $\hat{A}$ is the normalized adjacency matrix, and $W^{\left(l\right)}$ is the trainable weight matrix of the l-th GCN layer.

Among them, $H^{\left(\text{0}\right)}=X$, and $W^{\left(l\right)}$ denotes the learnable weight matrix of thel-th layer. The matrix $\hat{A}=D^{-\frac{\text{1}}{\text{2}}}\left(A+I\right)D^{-\frac{\text{1}}{\text{2}}}$ is the normalized adjacency matrix, where D is the diagonal degree matrix of A, I is the identity matrix, and relu(·represents the ReLU activation function. After l layers of GCN processing, the feature matrix of the drug molecular graph is $H_{d}=H^{\left(L\right)}\in R^{n\times h}$, where h is the hidden layer dimension. Because different drug molecular graphs may have different numbers of atoms, we apply a global max pooling operation to $H_{d}$ so as to obtain drug feature vectors of uniform dimension. Specifically, we derive the global features $D_{i},D_{j}\in R^{\text{1}\times h}$ for drugs $d_{i}$ and $d_{j}$, respectively. For a drug combination$\left(d_{i},d_{j}\right)$, its feature representation is given by the sum of the two global drug features:


$$D_{ij}=D_{i}+D_{j},$$ (2)

where$D_{i}$ and $D_{j}$ are the feature vectors of drugs$D_{i}$ and$D_{j}$, respectively, and $D_{ij}$ denotes the feature representation of the drug combination.

The feature representation of cell line i is derived from the constructed cell-line-specific gene expression graph $G^{\left(i\right)}$. Similar to processing a drug molecular graph, the l-th GCN layer for the cell line graph is defined as:


$$H^{\left(l\right)}=ReLU\left(\hat{A_{sp}}H^{\left(l-\text{1}\right)}W^{\left(l\right)}\right),$$ (3)

where $H^{\left(\text{0}\right)}=C_{e}^{\left(i\right)}$, and $\hat{A_{sp}}=D^{-\frac{\text{1}}{\text{2}}}\left(A_{sp}+I\right)D^{-\frac{\text{1}}{\text{2}}}$is the normalized adjacency matrix. Here, $A_{sp}$ represents the topological structure of the cell line’s network, and D is the diagonal degree matrix of $A_{sp}^{\left(i\right)}$. After l GCN layers, the resulting feature matrix of cell linei is $C_{e}^{\prime (i)}=H^{\left(L\right)}\in R^{G_{n}\times h}$.

When a drug acts on a cell line, it usually affects target proteins, signaling pathways, and other biological processes, thereby inducing therapeutic or other biological effects. Consequently, when two drugs act jointly on a cell line, they necessarily influence gene expression. To capture this effect, we introduce a cross-attention mechanism to fuse the features of the drug combination with those of the cell line, thereby modeling the influence of the drug combination on cell-line gene expression.

In this study, the transposed drug combination feature $D_{ij}^{T}\in R^{h\times 1}$ serves as the key vector K, while the cell-line gene expression features $C_{e}^{\prime (i)}\in R^{G_{n}\times h}$ act as the query vector Q and value vector V. Here, $G_{n}$ denotes the number of genes in the cell line, and h is the dimension of the gene feature representation. By applying trainable weight matrices $W_{Q}$, $W_{K}$, and $W_{V}$, we obtain the following feature transformations:


$$Q=C_{e}^{\prime (i)}W_{Q},K=D_{ij}^{T}W_{K},V=C_{e}^{\prime (i)}W_{V},$$ (4)

with$K\in R^{h\times b}$ and $Q,V\in R^{G_{n}\times b}$, where b is the hidden-layer dimension. The attention weight matrix $\text{A}\in R^{G_{n}\times h}$ is computed by


$$A=softmax\left(\frac{Q*K^{T}}{\sqrt{b}}\right),$$ (5)

where* denotes the dot product. To maintain a consistent hidden-layer dimension, we use a linear layer to reduce the dimension of A to $R^{G_{n}\times b}$. According to Eq. (5), we then obtain the cell-line gene expression feature $V^{\prime }$, which incorporates drug-combination effects:


$$V^{\prime }=A⊙V,$$ (6)

where ⊙ denotes the Hadamard product. Additionally, to mitigate the vanishing-gradient problem, we employ a residual strategy by concatenating $V^{\prime }$ and $C_{e}^{\prime (i)}$. This yields a cell-line feature representation $C_{re}$ that accounts for drug-combination effects:


$$C_{re}=Concat\left(V^{\prime },C_{e}^{\prime (i)}\right),$$ (7)

where $V^{\prime }\in R^{G_{n}\times b}\text{and}C_{e}^{\prime (i)}\in R^{G_{n}\times h}$. Hence, $C_{re}\in R^{G_{n}\times (b+h)}$. Finally, we apply global average pooling followed by a transpose operation to obtain the cell-line feature representation $C_{re}\in R^{\text{1}\times G_{n}}$. Thus, the fused network capturing the influence of the drug combination on gene expression in cell line i is given by


$$G^{\prime (i)}=\left(C_{re}^{\left(i\right)},A_{sp}^{\left(i\right)}\right),$$ (8)

### 4.4 Causal attention-based module for drug combination synergy prediction

In order to accurately identify cell-line-specific synergistic drug combinations and provide interpretable analyses of the underlying molecular mechanisms, this study proposes a graph decoupling method based on a causal attention mechanism (Fig 1(c)). Through this method, the fused network $G^{\prime }$, which encapsulates the effects of drugs on cell-line gene expression, is decoupled into a causal subgraph and a shortcut subgraph. The causal subgraph represents the true synergistic interaction of the drug combination on the cell line, while the shortcut subgraph includes confounding factors (i.e., “shortcut features”) associated with the prediction. The benefit of decoupling for synergy prediction is that, by separating the true synergistic signals (causal features) from potential confounding factors (shortcut features), the model can focus on causal relationships that are crucial to synergy, thereby performing more stable and accurate synergy predictions.

**Soft mask estimation.** To effectively decouple the causal subgraph and the shortcut subgraph, we employ an attention mechanism and introduce soft masks for both nodes and edges. For a given fused network $G^{\prime }$ of drug combination and cell-line gene expression features, we construct two multilayer perceptrons (MLPs) to assess attention scores separately from the perspectives of nodes and edges. Equations (9) and (10) respectively provide the causal and shortcut attention scores for node ${\text{v}}_{i}$ and for the edge $\left({\text{v}}_{i},{\text{v}}_{j}\right)$:


$$\alpha_{c_{i}},\alpha_{s_{i}}=\sigma \left({\text{MLP}}_{\text{node}}({𝐡}_{i})\right),$$ (9)

$$\beta_{c_{ij}},\beta_{s_{ij}}=\sigma \left({\text{MLP}}_{\text{edge}}({𝐡}_{i}∥{𝐡}_{j})\right),$$ (10)

where σ(·) is the softmax function, $h_{i}$ is the feature vector of node $v_{i}$, and``∥"denotes the concatenation operator; hence, $\left({\text{v}}_{i},{\text{v}}_{j}\right)$ is the feature vector corresponding to the edge $\left({\text{v}}_{i},{\text{v}}_{j}\right)$. The causal and shortcut attention scores satisfy $\alpha_{c_{i}}+\alpha_{s_{i}}=\text{1}$ and $\beta_{c_{ij}}+\beta_{s_{ij}}=\text{1}$, indicating that the attention scores for the causal subgraph and the shortcut subgraph are complementary in this study.

Based on the calculated node and edge attention scores $\alpha_{c_{i}}$,$\beta_{s_{ij}}$, we construct matrices $M_{x}$ and $M_{a}$ as soft masks for the causal subgraph. Meanwhile, $\alpha_{s_{i}}$and $\beta_{s_{ij}}$ are used to construct ${\overset{―}{M}}_{x}=\text{1}-M_{x}$ and ${\overset{―}{M}}_{a}=\text{1}-M_{a}$ as soft masks for the shortcut subgraph. We then apply these soft masks to the graph structure via the Hadamard product to obtain the causal subgraph $G_{c}$ and the shortcut subgraph $G_{s}$:


$$G_{c}=\left(\;\;\;\begin{array}{c} A_{sp}^{\left(i\right)}⊙M_{a},C_{re}^{\left(i\right)}⊙M_{x} \end{array}\;\;\;\right),$$ (11)

$$G_{s}=\left(A_{sp}^{\left(i\right)}⊙{\overset{―}{M}}_{a},C_{re}^{\left(i\right)}⊙{\overset{―}{M}}_{x}\right),$$ (12)

where $A_{sp}^{\left(i\right)}$ is the adjacency matrix of the cell-line-i-specific PPI network, $C_{re}^{\left(i\right)}$ is the node feature matrix that integrates both the drug-combination and cell-line gene expression features, and ⊙ denotes the Hadamard product.

**Graph decoupling**. Having obtained the causal subgraph $G_{c}$ and the shortcut subgraph $G_{s}$at both the node and edge levels, we now decouple the cell-line-specific PPI network subgraphs to extract the corresponding causal and shortcut features. Specifically, we apply a graph neural network (GCN) and a readout model to $G_{c}$ and $G_{s}$, respectively, to obtain their global vector representations:


$${𝐡}_{G_{c}}=f_{\text{readout}}\left(\text{GCN}(G_{c})\right),$$ (13)

$${𝐡}_{G_{s}}=f_{\text{readout}}\left(\text{GCN}(G_{s})\right),$$ (14)

where ${𝐡}_{G_{c}}$is the causal feature extracted from ${𝐡}_{G_{c}}$, and ${𝐡}_{G_{s}}$ is the shortcut feature extracted from $G_{s}$. We then concatenate the drug combination feature $D_{ij}$ with the causal feature ${𝐡}_{G_{c}}$, and use a classifier $\Phi_{c}$ to predict the probability of synergy:


$${𝐳}_{G_{c}}=\Phi_{c}\left(D_{ij}∥{𝐡}_{G_{c}}\right),$$ (15)

where ${𝐳}_{G_{c}}$ is the predicted synergy probability. We employ the cross-entropy loss for training:


$$L_{causal}=-\frac{\text{1}}{\left|D\right|}\sum_{G\in D}\;{𝐲}_{G}^{⊤}\text{log}({𝐳}_{G_{c}}),$$ (16)

where D is the training dataset and ${𝐲}_{G}$ is the ground truth label for each drug combination.

Regarding the shortcut features ${𝐡}_{G_{s}}$ from the shortcut subgraph, we desire them not to affect the true classification outcome. Hence, we randomly generate a label ${𝐲}_{\text{rand}}$ as the target distribution, use a classifier $\Phi_{s}$ to predict the corresponding synergy score ${\text{z}}_{G_{c}}=\Phi_{c}\left({\text{h}}_{G_{s}}\right)$, and then minimize the KL-divergence between ${𝐳}_{G_{s}}$ and ${𝐲}_{\text{rand}}$:


$$L_{bias}=\frac{\text{1}}{\left|D\right|}\sum_{G\in D}\;KL\left({𝐲}_{\text{rand}},{𝐳}_{G_{s}}\right),$$ (17)

By optimizing these two objectives, we can effectively disentangle causal features from shortcut features. Because of the presence of noise and shortcut features, the mutual information between the causal features and the labels is higher than that between the full graph and the labels, preventing the causal subgraph from degenerating into the complete, undecoupled graph.

### Causal attention-based module for drug combination synergy prediction

Because confounding effects may influence the final results, we employ the backdoor adjustment strategy from causal inference theory to remove biases introduced by confounders. The backdoor adjustment is an effective solution to mitigate confounding effects by stratifying the shortcut features and pairing the causal features with each stratification of shortcut features to form an ``intervened graph.“ However, due to the irregular structure of graph data, it is challenging to perform such interventions directly at the data level. To address this, we adopt a causal attention strategy that avoids this issue by performing implicit interventions at the representation level, guided by a loss function derived from backdoor adjustment. Concretely, as shown in Eqs. (18) and (19):


$${𝐳}_{G^{\prime }}=\Phi \left(D_{ij}∥({𝐡}_{G_{c}}+{𝐡}_{G_{s^{\prime }}})\right),$$ (18)

$$L_{interv}=-\frac{\text{1}}{\left|𝒟|\cdot |\hat{T}\right|}\sum_{G\in D}\;\sum_{t^{\prime }\in \hat{T}}\;{𝐲}_{G}^{⊤}\text{log}({𝐳}_{G^{\prime }}),$$ (19)

where ${𝐡}_{G_{c}}$ is the causal feature obtained from Eq. (13), and ${𝐡}_{G_{s^{\prime }}}$is the feature representation of the shortcut subgraph $G_{s^{\prime }}$ from a different stratification, obtained via Eq. (14). The vector $D_{ij}$ is the feature representation of the drug combination $D_{i}$,$D_{j}$. By combining these representations, we simulate the model’s predictions under varying shortcut features. The output ${𝐳}_{G^{\prime }}$ is the prediction result of the classifier Φ on the “implicit intervened graph,” and $\hat{T}$ is the set of strata for the estimated shortcut subgraphs, encompassing various shortcut features in the training data. In practice, we use random addition to implement the intervention in Eq. (18). Random addition simulates different “scenarios” by adding the causal feature representation to randomly selected shortcut feature representations, thereby creating a new, combined graph representation. This approach helps the model maintain stable predictions when confronted with different shortcut features. We refer to Eq. (19) as the causal intervention loss $L_{interv}$. It enforces consistent and stable predictions across different shortcut stratifications because they share the same causal features.

Finally, the objective function of our CASynergy algorithm is defined as the weighted sum of these losses:


$$L=L_{causal}+\lambda_{\text{1}}L_{bias}+\lambda_{\text{2}}L_{interv},$$ (20)

where $\lambda_{\text{1}}$ and $\lambda_{\text{2}}$ are hyperparameters that determine the strengths of the decoupling and causal intervention, respectively. The total loss L consists of three parts: $L_{causal}$ is the supervised classification loss based on causal features, ensuring that the model correctly predicts the labels; $L_{bias}$ encourages the shortcut features to produce uniformly distributed predictions without bias toward any particular category; and $L_{interv}$ ensures consistent predictions under different shortcut features, reinforcing the underlying causal relationships.

By leveraging the causal attention mechanism, the CASynergy algorithm is capable of making robust predictions without relying on specific shortcut features. This also endows the model with strong generalization capabilities when handling test data drawn from distributions different from those of the training data.

### 4.5 Performance measures

To evaluate the effectiveness of CASynergy, we divided the datasets into training, validation, and testing sets with an 8:1:1 ratio. The training and validation sets underwent five- fold cross-validation, and the results on the independent test set were averaged to obtain the final outcomes. To comprehensively describe the performance of the prediction model, we employed common classification metrics, including Area Under the Receiver Operating Characteristic Curve (AUC-ROC), Area Under the Precision-Recall Curve (AUC-PR), Accuracy (ACC), and F1 Score. These metrics respectively reflect the model’s ability to distinguish between positive and negative samples, balance precision and recall, overall prediction accuracy, and comprehensive performance.

In the specific experiments, to identify the optimal hyperparameter configuration, we conducted hyperparameter optimization using grid search. This optimization included searching for learning rates within the set {10^−2^, 10^−3^, 10^−4^, 10^−5^}, batch sizes within{16, 32, 64, 128, 256}, cross-attention layers within {1, 2, 3},and testing both Global Max Pooling and Global Average Pooling for global pooling. By comparing the experimental results of different hyperparameter combinations in the validation set, we ultimately selected a set of parameter configurations that achieved the best performance on the independent test set, as shown in Tables S2 and S3 in the appendix.

## Supporting information

S1 Table summarizes the OncologyScreen dataset, comprising 28 cancer cell lines from CCLE and GEO, spanning skin, ovary, large intestine, lung, breast, and prostate tissues. Each entry lists the cell line name, tissue origin, and unique DepMap ID, enabling integration with multi-omics data and cross-study analyses.
(XLSX)

S2 Table lists the hyperparameters optimized for the CASynergy model, including learning rates (10 ⁻ ² to 10 ⁻ ⁵), batch sizes (16–156), cross-attention layers (1–3), and global pooling methods (Max/Average). The final configuration was empirically determined through systematic validation.
(XLSX)

S3 Table presents the hyperparameter tuning results.
(XLSX)

S4 Table CASynergy performance under random gene expression masking (0–30%): AUC, AUPR, F1, and ACC (mean ± SD) evaluated with five-times five-fold cross-validation. (XLSX)

S5 Table Data-efficiency analysis on Oncology-Screen: CASynergy performance versus training-set size (30%, 50%, 70%) under five-times five-fold cross-validation (mean ± SD). (XLSX)

S6 Table Multi-omics augmentation on Oncology-Screen: CASynergy performance across feature configurations (expression only; + mutation; + CNV; + mutation+CNV) under five-times five-fold cross-validation (mean ± SD). (XLSX)

S1 Fig Gene distribution probability density graphs. (TIFF)
