# Supplementary figures and images for: CASynergy: A causal attention model for interpretable prediction of cancer drug synergy
Source: PLoS Comput Biol. 2025 Oct 15;21(10):e1013567. doi: 10.1371/journal.pcbi.1013567 (PMC12548910; doi:10.1371/journal.pcbi.1013567)

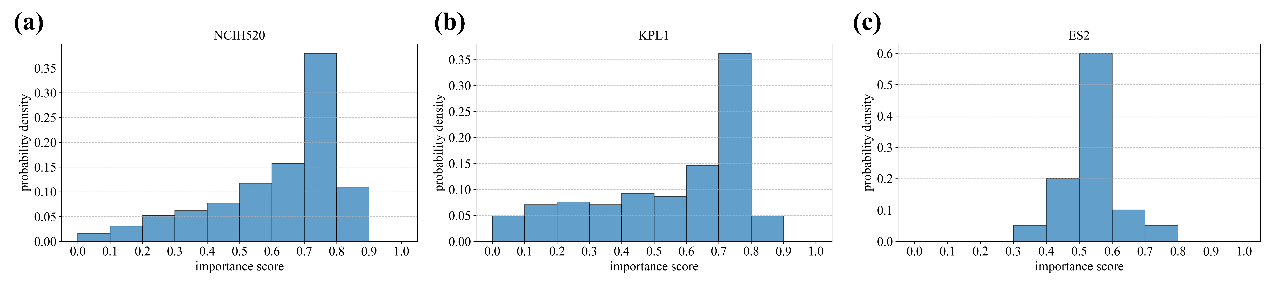

Supplement: S1 Fig — (TIFF) [file pcbi.1013567.s007.tiff]
